# The complete mitochondrial genome of white-tailed mole (*Parascaptor leucura*)

**DOI:** 10.1080/23802359.2021.1899871

**Published:** 2021-03-19

**Authors:** Fei Xie, Dan Chen, Boxin Qin, Changkun Fu, Shunde Chen

**Affiliations:** College of Life Sciences, Sichuan Normal University, Chengdu, PR China

**Keywords:** Bayesian phylogenetic tree, Talpidae, mitogenome structure

## Abstract

The white-tailed mole (*Parascaptor leucura*) belongs to genus *Parascaptor*, which is a monotypic genus distributed across Southwestern China, Assam (India), Bengal, and Northern Burma, and Laos. In this study, we obtained the complete mitochondrial genome of *Parascaptor leucura*. The genome is total 16,875 bp in length, containing 13 protein-coding genes, 22 transfer *RNA* genes (tRNA), two ribosomal *RNA* genes (rRNA), and two non-coding regions, with a base composition of 33.5% A, 26.4% T, 25.7% C, and 14.3% G. The nucleotide sequence data of 13 protein-coding genes of *P. leucura* and other nine Eulipotyphla species were used to reconstruct a Bayesian phylogenetic tree. The tree shows that *P. leucura* belongs to subfamily Talpinae and is closely related to *Scaptochirus moschatus*.

*Parascaptor leucura* (Blyth 1850) belongs to subfamily Talpinae. Its type locality is Mount Assam Kashgar, India. The species typically occurs in mountain forests and scrub grasslands at elevations of 1000–3000 locality (Hoffmann and Lunde 2009). *P. leucura* lacks a pair of small upper premolars compared with other species of Talpinae, so Gill established the genus *Parascaptor* (Gill 1875). This view was endorsed by most scholars (Corbet 1978; Abe et al. 1991; Hutterer 1993, 2005). Subsequently, the *P. leucura* is considered to be the most basic group of the Talpini based on morphology, and further proved the effectiveness of the genus *Parascaptor* (Sanchez-Villagra et al. 2006). Here, we sequenced the complete mitogenome of *P. leucura* and restructured Bayesian phylogenetic tree with other nine Soricomorpha species.

The individual was captured in Muli County, Sichuan Province, China(Latitude: 28°0'31"N, Longitude: 101°7'22"E; H: 3545 m) captured in Muli County, Sichuan Province, China The specimen was deposited at Sichuan Academy of Forestry (Shaoying Liu, email: shaoyliu@163.com) under the voucher number SAF12011. The total DNA of *P. leucura* was extracted by TRIzol^®^ Reagent, and deposited at the College of Life Sciences, Sichuan Normal University. The mitogenome was sequenced using the Illumina Hiseq 4000 sequencing platform. The library was constructed by nano DNA sample prep Kit, and DNA was broken to 300–500s sequenced using theAdding A at 3′end and connecting the joint, after making up the flat, eight cycles were amplified by PCR. 2 * 150no DNA sample prep Kit, and DNA was broken to 300HorovitzI, MotokawaM. 2006. Aersion 3.10.1 (Nurk et al. 2013). These high coverage depth and long assembly length sequences were selected as candidate sequences. The mitochondrial scaffold sequence was confirmed by NCBI. According to the paired-end and overlap relationship of reads, compare the clean reads back to the assembled Scaffolds, to fill and optimize the assembly results by GapClos er (version 1.12). The final mitochondrial genome sequence was obtained by the reference genome, to get the calibration result by comparing the start position and orientation of mitochondrial assembly sequence. Annotate the complete mitochondrial genome using MITOS (Bernt et al. 2013).

The complete mitochondrial genome of *P. leucura* is 16,875 bp, which contains 13 protein-coding genes, two ribosomal *RNA* genes (rRNA), 22 transfer *RNA* genes (tRNA), and two non-coding regions including one light strand replication origin (OL), and one non-coding region (D-Loop). The total length of 13 protein-coding genes is 11,370 bp, and all of the protein-coding genes begin with ATG, except for ND2, ND3, ND6 (ATA), and ND5 (ATT). Termination codons of eight protein-coding genes (COX1, COX2, ATP8, ATP6, ND4L, ND1, ND5, and ND6) are TAA, termination codons of three protein-coding genes (ND3, COX3, and ND4L) are incomplete, and termination codons of Cyt *b* and ND2 are AGA and TAG, respectively. The entire base composition is as follows: 32.5% A, 26.4% T, 25.7% G, and 14.3% C.

So as to explore the evolution of family Talpidae, thirteen concatenated mitochondrial protein genes from *P. leucura* and other nine species were used for phylogenetic analysis. Two species of Soricidae were used as outgroups. We used BEAST version 1.6.1 (Drummond et al. 2012) for Bayesian phylogenetic reconstructions, and the chain length was set at 100 million generations with the sampling of every 5000 generation. The best-fit GTR+I+G model of DNA substitution was selected using Akaike Information Criterion (AIC) test in JModelTest 2 (Darriba et al. 2012).

The Bayesian phylogenetic tree is divided into two groups: Soricidae and Talpidae, and *P. leucura* is sister to *Scaptochirus moschatus* with well-supported value (BPP = 1.00) (Figure 1).

**Figure 1.**
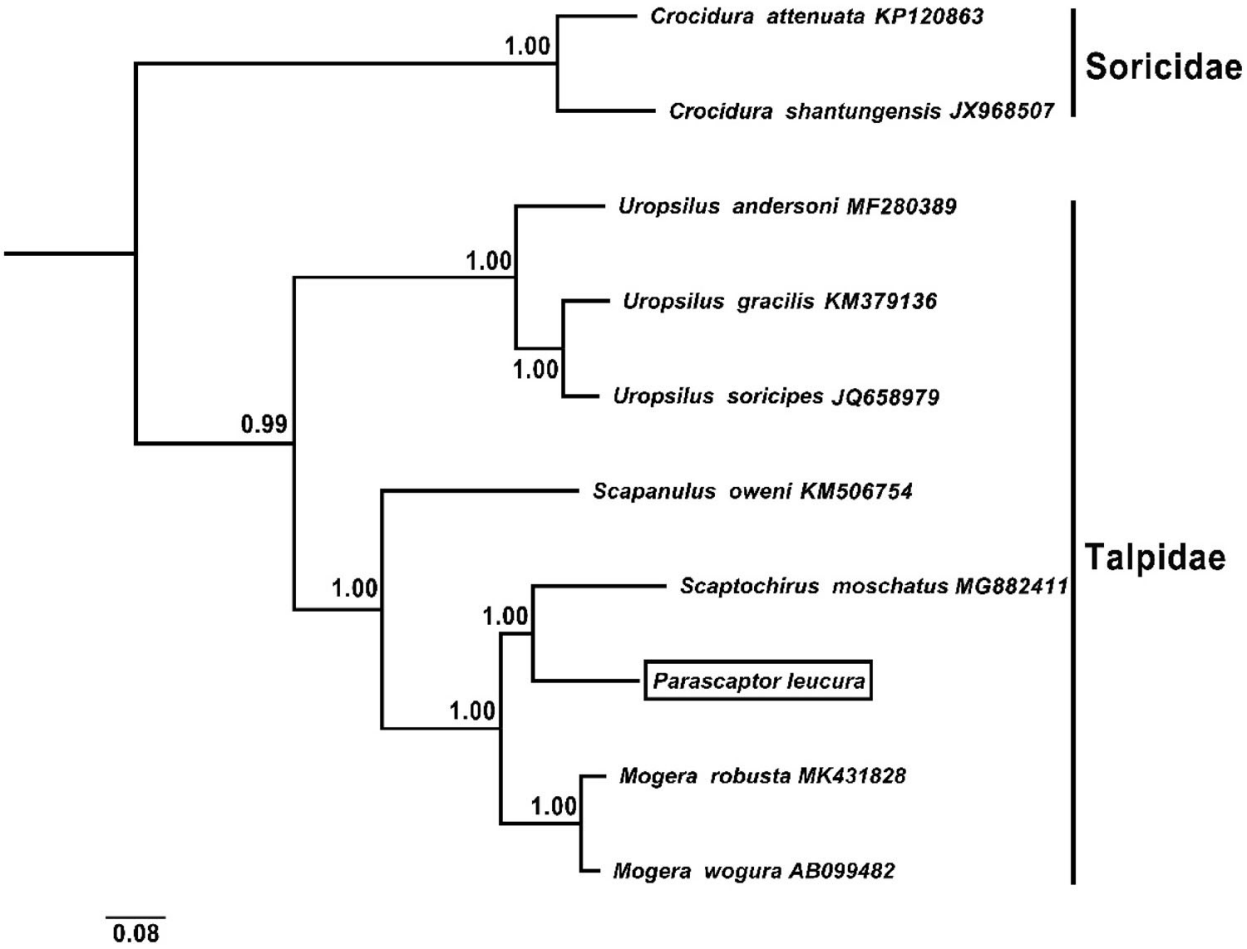
Bayesian phylogenetic tree based on 13 protein genes of mitochondrial genome. Numbers by the nodes indicate Bayesian posterior probabilities.

## Data Availability

The genome sequence data that support the findings of this study are openly available in GenBank of NCBI at (https://www.ncbi.nlm.nih.gov/) under the accession no. MW114662. The associated BioProject, SRA, and Bio-Sample numbers are PRJNA694492, SUB8933094, and SAMN17525985, respectively.
